# P-241. Epidemiology of ventilator-associated pneumonia among patients requiring extracorporeal membrane oxygenation (ECMO): a retrospective cohort study

**DOI:** 10.1093/ofid/ofae631.445

**Published:** 2025-01-29

**Authors:** Caden Nowak, Owen Albin

**Affiliations:** University of Michigan, Ann Arbor, Michigan; University of Michigan Medical School, Ann Arbor, MI

## Abstract

**Background:**

Ventilator-associated pneumonia (VAP) is a major cause of morbidity and mortality among critically ill patients requiring extracorporeal membrane oxygenation (ECMO), yet the epidemiology of VAP in this patient cohort remains uncertain, particularly following the COVID-19 pandemic. The aim of this study is to characterize the epidemiology and microbiologic causes of VAP in patients requiring ECMO at a tertiary care academic referral center following COVID-19 (2020-23). Here, we present preliminary study data for the year 2020.

**Figure 1.**
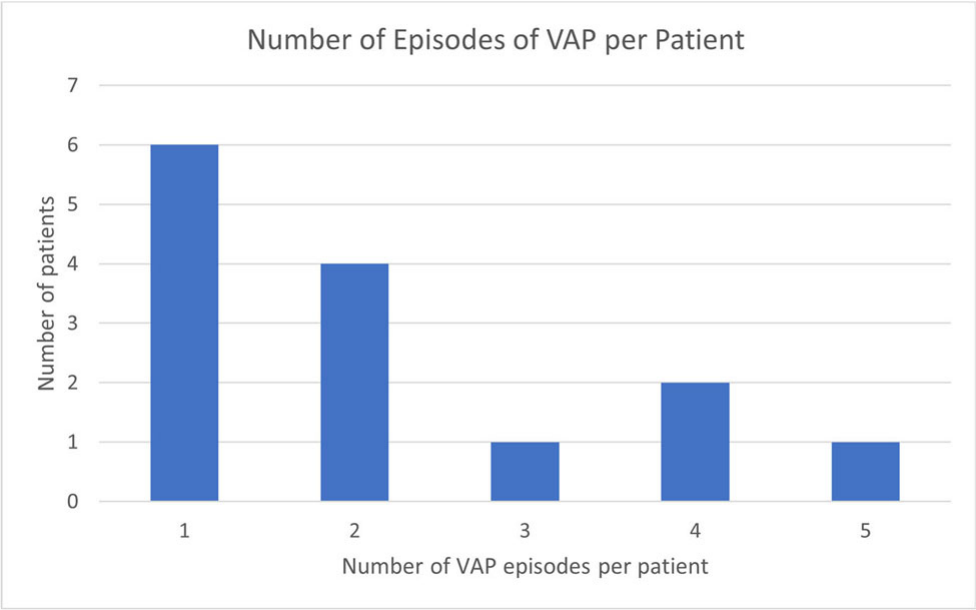
Histogram showing number of VAP episodes per patient among 14 ECMO patients who experienced VAP.

**Methods:**

Retrospective cohort study of adult patients (age ≥ 18) hospitalized at Michigan Medicine University Hospital in calendar year 2020 who required use of ECMO (venoarterial or venovenous) during hospitalization. Patient demographic characteristics, medical comorbidities, and microbiologic data were abstracted from the electronic medical record via a structured query. VAP was defined as occurrence of a positive quantitative respiratory culture obtained via distal lung sampling.

**Figure 2.**
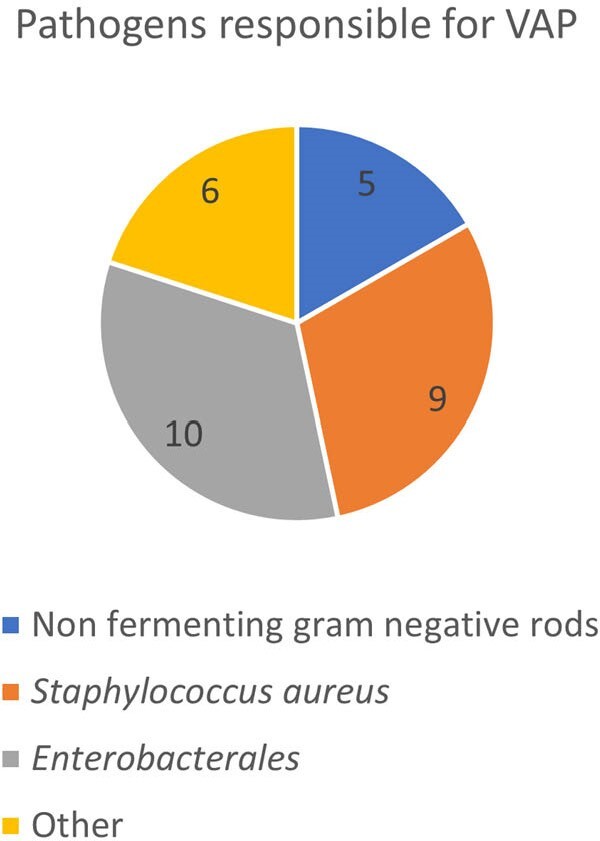
Pathogens isolated from 30 positive quantitative respiratory cultures obtained in 14 ECMO patients in 2020.

**Results:**

102 adult patients required use of ECMO in 2020. The mean patient age was 50.3 (SD 14.3) years of age. 61 (59%) of patients were male. 64% of patients were Caucasian, and 21% were African-American. Relevant patient comorbidities included congestive heart failure (67%), liver disease (44%), chronic pulmonary disease (43%), and diabetes (36%). 14 (14%) of patients experienced VAP. Of these patients who experienced VAP, 8 (57%) experienced >1 episode of VAP (Figure 1), and 7 (50%) died prior to hospital discharge. Figure 2 illustrates the microbiologic causes of VAP in this patient cohort.

**Conclusion:**

In a tertiary referral academic medical center, a minority of ECMO patients experienced the vast majority of VAP cases and suffered high rates of in-hospital mortality. *Enterobacteriales* and *Staphylococcus aureus* were responsible for the majority of VAP cases among patients requiring ECMO. Future work will extend these analyses through the year 2023 and capture antimicrobial use rates.

**Disclosures:**

**All Authors**: No reported disclosures

